# hTERT Immortalization Stabilizes Human Umbilical Cord MSCs and Maintains Their Small Extracellular Vesicles With Preserved Immunomodulatory Activity and Primordial Follicle‐Activating Capacity

**DOI:** 10.1111/acel.70681

**Published:** 2026-08-21

**Authors:** Yating Chen, Wei Liu, Ying Tian, Xinyu Wang, Haonan Fan, Mingyu Yang, Feiyang Diao, Jing Li

**Affiliations:** ^1^ State Key Laboratory of Reproductive Medicine and Offspring Health Nanjing Medical University Nanjing Jiangsu P. R. China; ^2^ Reproductive Medicine Center, Tongji Hospital, Tongji Medical College Huazhong University of Science and Technology Wuhan China; ^3^ The Center for Clinical Reproductive Medicine, State Key Laboratory of Reproductive Medicine and Offspring Health The First Affiliated Hospital of Nanjing Medical University Nanjing Jiangsu China; ^4^ Changzhou Medical Center Nanjing Medical University Changzhou Jiangsu China

**Keywords:** hTERT immortalization, macrophage polarization (M1/M2), primordial follicle activation, sEVs

## Abstract

Cellular senescence is a fundamental hallmark of aging and represents a major barrier to the scalable and reproducible application of mesenchymal stromal cell (MSC)‐derived small extracellular vesicles (sEVs). Senescent human umbilical cord mesenchymal stromal cells (hUCMSCs) exhibit impaired proliferative capacity, disrupted mitochondrial homeostasis, and altered secretory phenotypes, which may compromise the biological activity and therapeutic reliability of sEVs. Here, we investigated whether overexpression of human telomerase reverse transcriptase (hTERT) attenuates senescence‐associated deterioration and stabilizes sEV functional properties. Late‐passage hUCMSCs displayed canonical senescence features and mitochondrial dysfunction, all of which were markedly alleviated by hTERT expression. Functionally, sEVs derived from senescent cells exhibited impaired immunomodulatory activity, whereas sEVs from hTERT‐expressing cells largely restored this function. Mechanistically, senescence was associated with altered sEV cargo, including enrichment of miR‐217‐5p, which contributed to diminished immunomodulatory potency, at least in part through modulation of SIRT1‐associated inflammatory signaling in recipient macrophages. Proteomic profiling further showed that H11‐sEVs retained a young‐like protein cargo profile, particularly for proteins associated with immune and inflammatory regulation. In parallel, sEVs from all three groups retained primordial follicle‐activating capacity, consistent with the broad retention of PI3K‐Akt pathway‐related proteins in their proteomic profiles and PI3K‐Akt pathway activation in ovarian tissues. In aged female mice, H11‐sEVs exhibited young‐like ovarian protective activity. Collectively, these findings demonstrate that hTERT uncouples cellular senescence from sEV functional decline, supporting the development of potency‐stabilized sEV sources for aging‐related and regenerative applications and providing insight into the biological roles of senescent cell‐derived sEVs.

## Graphical abstract

hTERT immortalization stabilizes hUCMSC‐derived sEV function by alleviating cellular senescence and preserving young‐like cargo profiles. H11‐sEVs retain immunomodulatory activity, primordial follicle‐activating capacity, and ovarian protective effects.

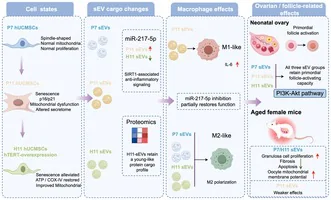

## 1 Introduction

For decades, mesenchymal stem cells (MSCs) have attracted substantial scientific interest due to their accessibility, immunomodulatory capacity, and therapeutic potential in regenerative medicine across both experimental and clinical settings (de Witte et al. 2018; Galleu et al. 2017; Patel et al. 2013). Compared with embryonic stem cells or induced pluripotent stem cells, MSCs are generally regarded as a safer option for stem cell–based therapies because of their lower risk of tumorigenicity (Jung et al. 2012). Among the various MSC sources, human umbilical cord‐derived mesenchymal stem cells (hUCMSCs) stand out as an appealing and ethically acceptable choice (Shaikh et al. 2022). They can be readily isolated and expanded in vitro, demonstrate stable biological properties, and exhibit low immunogenicity and tumorigenic potential. Moreover, obtaining MSCs from umbilical cords raises no ethical controversy, causes no discomfort to donors, and poses minimal risk of disease transmission within the population (Mebarki et al. 2021). Taken together, hUCMSCs represent a promising MSC source for the treatment of a wide range of diseases.

Despite the many advantages of hUCMSCs, their clinical translation still faces substantial challenges. A major barrier is the need to obtain high‐quality, stable, and scalable cells suitable for therapeutic manufacturing. During in vitro expansion, MSCs inevitably undergo replicative senescence, a stress‐induced state marked by cell‐cycle arrest and reduced proliferative capacity. Senescence also compromises key MSC functions, including self‐renewal, differentiation potential, and immunomodulatory activity, ultimately diminishing their therapeutic efficacy (Li et al. 2017). To overcome these limitations, numerous strategies have been explored to delay or prevent cellular aging. The SV40 large T antigen (SV40T), human telomerase reverse transcriptase (hTERT), and human papillomavirus E6/E7 genes have all been employed to achieve MSC immortalization (Kim et al. 2024; Wang et al. 2021). Telomerase, a ribonucleoprotein complex composed of hTERT, hTR, and associated factors, elongates telomeres via reverse transcription, thereby counteracting telomere attrition during cell division. Because telomere shortening is a key driver of MSC replicative senescence in vitro (Wang et al. 2021), telomerase activation and telomere extension have emerged as effective anti‐aging approaches. hTERT‐immortalized MSCs have undergone progressive evaluation, including lifespan extension (Mihara et al. 2003), stable genetic engineering (Liu et al. 2013), safety assessment (Liang et al. 2012), and studies of manufacturability and preclinical performance. As a result, they now represent an important platform for standardized MSC research and cell‐material production. However, key concerns remain regarding long‐term genomic stability and safety. Although hTERT alone has a relatively low propensity to initiate tumorigenesis, extended culture periods increase the likelihood of accumulating cancer‐associated mutations, with the degree of risk heavily influenced by culture conditions (Serakinci et al. 2004). Additionally, the introduction of immortalizing genes carries inherent risks of genetic alterations and potential malignant transformation during prolonged cultivation. Therefore, before these technological advances can be widely and safely implemented in clinical practice, it is essential to further address safety challenges, standardize manufacturing protocols, and rigorously verify the reproducibility and therapeutic consistency of immortalized MSC products (Yamaguchi et al. 2024), In this study, P7 hUCMSCs were selected as a relatively young and stable expanded passage suitable for reproducible sEV production, while P11 hUCMSCs were used as a senescence‐associated late‐passage model. This classification was based on our observations of senescence‐related phenotypes in P11 cells and previous reports showing that WJ‐MSCs below passage 10, including P7, retain relatively stable biological characteristics, whereas P11 hUCMSCs already display passage‐associated functional decline (Ding et al. 2025; Gu et al. 2016; Lian et al. 2016).

In contrast to MSCs, small extracellular vesicles (sEVs) derived from MSCs have increasingly emerged as a promising cell‐free therapeutic modality due to their enhanced safety profile, ease of storage and transport, and convenient administration routes. sEVs are double‐membrane vesicles, typically 30–150 nm in diameter, released by most cell types into various biological fluids. They carry a diverse array of bioactive cargos, including proteins, nucleic acids, and microRNAs (Keshtkar et al. 2018), allowing them to exert biological functions similar to their parental MSCs and representing a potent alternative to cell‐based therapy. MSC‐sEVs have demonstrated therapeutic efficacy in a broad spectrum of conditions, including inflammatory disorders (Feng et al. 2025), tissue injuries (Nakazaki et al. 2023), cardiovascular diseases (Sun et al. 2020), and neurodegenerative diseases (Lin et al. 2024), as shown in both preclinical animal models and early‐phase clinical studies. However, accumulating evidence indicates that the composition and function of sEVs are influenced by the physiological state of their parental MSCs. For instance, MSC‐derived sEVs display notable age‐dependent differences in immune regulatory profiles (Fafián‐Labora et al. 2017). Senescent, late‐passage MSCs secrete greater numbers of smaller microvesicles (MVs) compared with early‐passage cells, and the proportion of CD105^+^ MSC‐MVs declines as MSCs undergo senescence (Lei et al. 2017). Despite these observations, systematic studies characterizing the functional differences between sEVs from young versus senescent MSCs are lacking. Consequently, most research and preclinical applications preferentially use sEVs derived from low‐passage MSCs (typically passages 7–9) to preserve potency and reproducibility, which, however, limits large‐scale production (Kou et al. 2022). To overcome these limitations, generating sEVs from immortalized MSCs represents a practical strategy to ensure consistent quality and long‐term availability. Specifically, hTERT‐immortalized MSCs allow continuous sEV production without concerns regarding cellular senescence or the safety risks of transplanting immortalized cells themselves. Several studies have shown that sEVs derived from hTERT‐immortalized MSCs retain therapeutic properties comparable to those of young MSCs (Tong et al. 2025); nevertheless, the mechanisms underlying this preservation remain largely unclear. Establishing standardized and scalable production methods for MSC‐sEVs is therefore essential to fully realize their therapeutic potential.

In this study, we generated hTERT‐overexpressing hUCMSCs and demonstrated their extended proliferative lifespan in culture. We observed that sEVs derived from proliferative P7 hUCMSCs and senescent P11 hUCMSCs differentially modulated macrophage polarization. Notably, hTERT overexpression restored the diminished anti‐inflammatory activity observed in P11‐derived sEVs. Furthermore, to our knowledge, this study provides the first evidence that hUCMSC‐derived sEVs from different cellular states retain primordial follicle‐activating capacity, and that hTERT‐rescued hUCMSC‐sEVs exhibit young‐like ovarian protective activity in aged mice. These functional findings were further supported by proteomic analysis, which revealed that H11‐sEVs retained a protein cargo profile more similar to young P7‐sEVs than to senescent P11‐sEVs. Our findings provide a new strategy for leveraging sEVs derived from hTERT‐immortalized MSCs for therapeutic applications.

## 2 Results

### 2.1 Altered Differentiation Ability in hUCMSCs During Long‐Term Culture

To investigate age‐associated alterations in hUCMSCs and their sEVs, primary hUCMSCs were first isolated. Surface marker expression was then assessed by flow cytometry according to the International Society for Cellular Therapy (ISCT) criteria (Salem and Thiemermann 2010). Markers were defined as positive when expressed by ≥ 95% of cells and negative when expressed by ≤ 2% of cells. The isolated cells exhibited high expression of canonical mesenchymal stem cell markers, including CD90, CD73, and CD105, whereas hematopoietic and endothelial markers such as CD19, HLA‐DR, CD11b, CD45, CD34, and CD31 were absent or expressed at very low levels (Figure S1A). These results confirm that the isolated cells fulfilled the ISCT criteria for hUCMSCs. To evaluate whether hUCMSCs maintained their multilineage differentiation potential during serial passaging, osteogenic and adipogenic induction were performed on cells from passages 7–11 (P7–P11). Staining analyses demonstrated that hUCMSCs at all examined passages were capable of differentiating into adipocyte‐like cells. In contrast, osteogenic differentiation capacity progressively declined with increased passage number. This loss of osteogenic potential in later‐passage cells indicates that cellular senescence disrupts the balance between osteogenic and adipogenic lineage commitment, with osteogenesis being disproportionately impaired (Figure S1B).

### 2.2 Altered Morphological and Growth Kinetics in hUCMSCs During the Sub—Culturing Process

**FIGURE 1:**
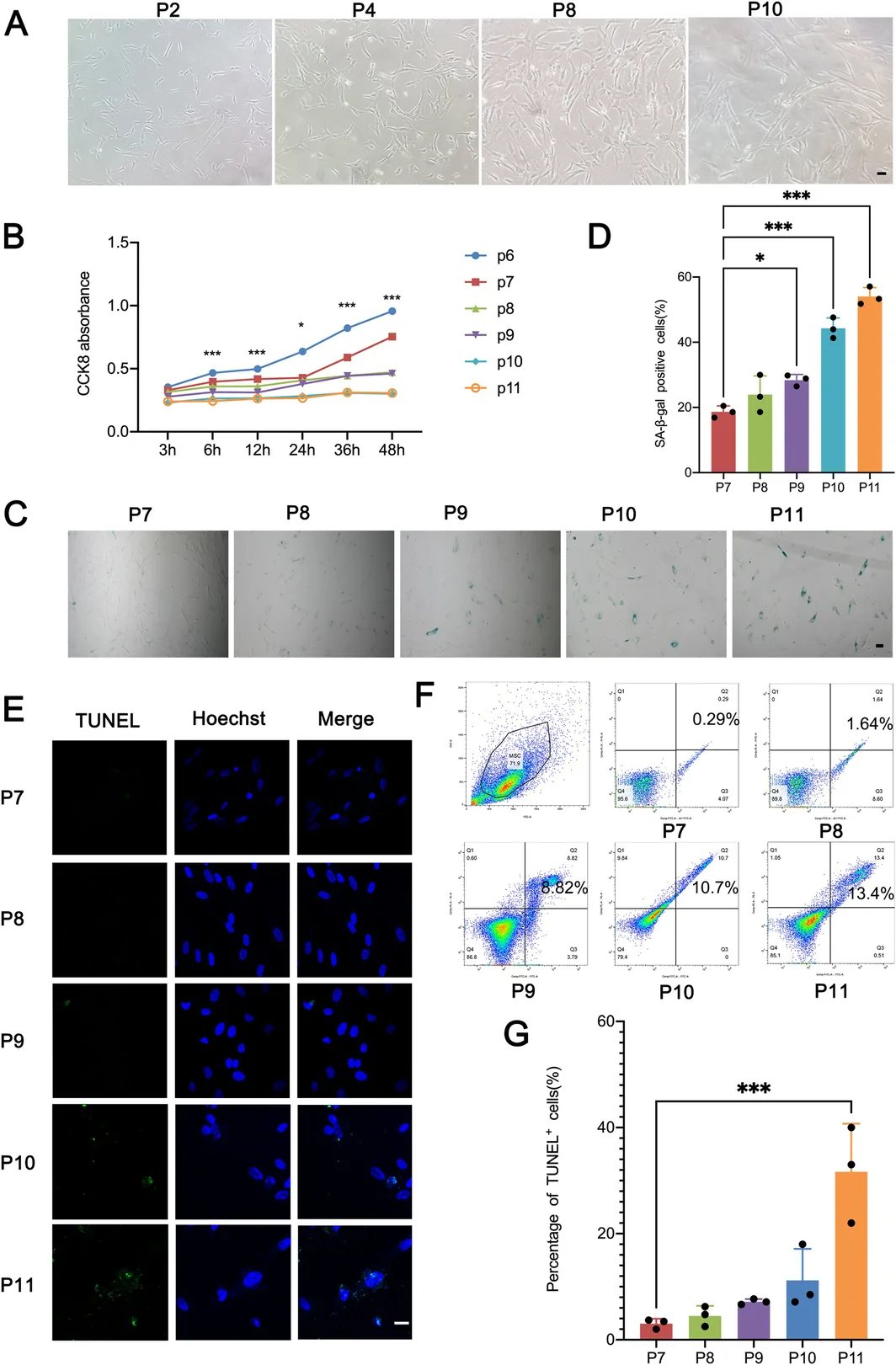
Morphological and functional assessments of cellular senescence and apoptosis in different experimental groups. (A) Morphological observations of cells in each group. (B) Growth curves showing the viability of six groups of cells. (C) β‐galactosidase staining of each group. (D) Quantification of β‐galactosidase‐positive cells. (E) TUNEL staining of five groups of cells. Green: TUNEL signal; blue: Hoechst 33342. (F) Flow cytometric analysis of apoptosis in five groups of cells with annexin V/propidium iodide staining. (G) Quantification of TUNEL‐positive cells. Data are expressed as mean ± SEM. *p < 0.05, ***p < 0.001. All scale bars = 20 μm.

hUCMSCs are widely used in tissue engineering and gene modification, yet their biological properties can shift during prolonged in vitro expansion (Gu et al. 2016; Truong et al. 2019). To characterize these changes, primary hUCMSCs were serially passaged and examined at passages 2, 4, 8, and 10. Early‐passage cells displayed a homogeneous spindle‐shaped morphology with smooth surfaces. By passage 10, the overall MSC‐like morphology was retained; however, a subset of cells exhibited elongated and flattened pseudopodia, accompanied by a marked reduction in proliferation (Figure 1A). Proliferation arrest became evident by passage 11 (Figure 1B). Senescence‐associated β‐galactosidase (SA‐β‐gal), a classical indicator of cellular senescence, was assessed across five passages. The proportion of SA‐β‐gal–positive cells increased progressively, with a pronounced accumulation of late‐senescent cells at higher passages (Figure 1C,D). We conducted qPCR analysis of the canonical senescence‐associated genes CDKN1A and CDKN2A, which encode p21 and p16, respectively. The results demonstrated that both CDKN1A and CDKN2A were upregulated in P11 relative to P7 (Figure S1C). These findings confirm that hUCMSCs undergo replicative senescence during long‐term culture. To determine whether senescence was accompanied by apoptosis, annexin V/propidium iodide staining was performed on cells from P7 to P11. Apoptosis increased steadily with passaging, and the late‐apoptotic population in P11 rose by approximately 13% relative to P7 (Figure 1F). TUNEL staining was also used to evaluate DNA breaks. Consistent with the flow cytometric apoptosis data, P11 cultures displayed a markedly higher proportion of TUNEL‐positive cells compared with P7 (Figure 1E,G). Furthermore, the expression level of γH2A.X protein in P11 was also higher than that in P7 (Figure S1D,E). These findings indicate that extended in vitro expansion of hUCMSCs promotes both apoptosis and DNA damage accumulation.

### 2.3 Establishment of hUCMSCs With hTERT Overexpression

**FIGURE 2:**
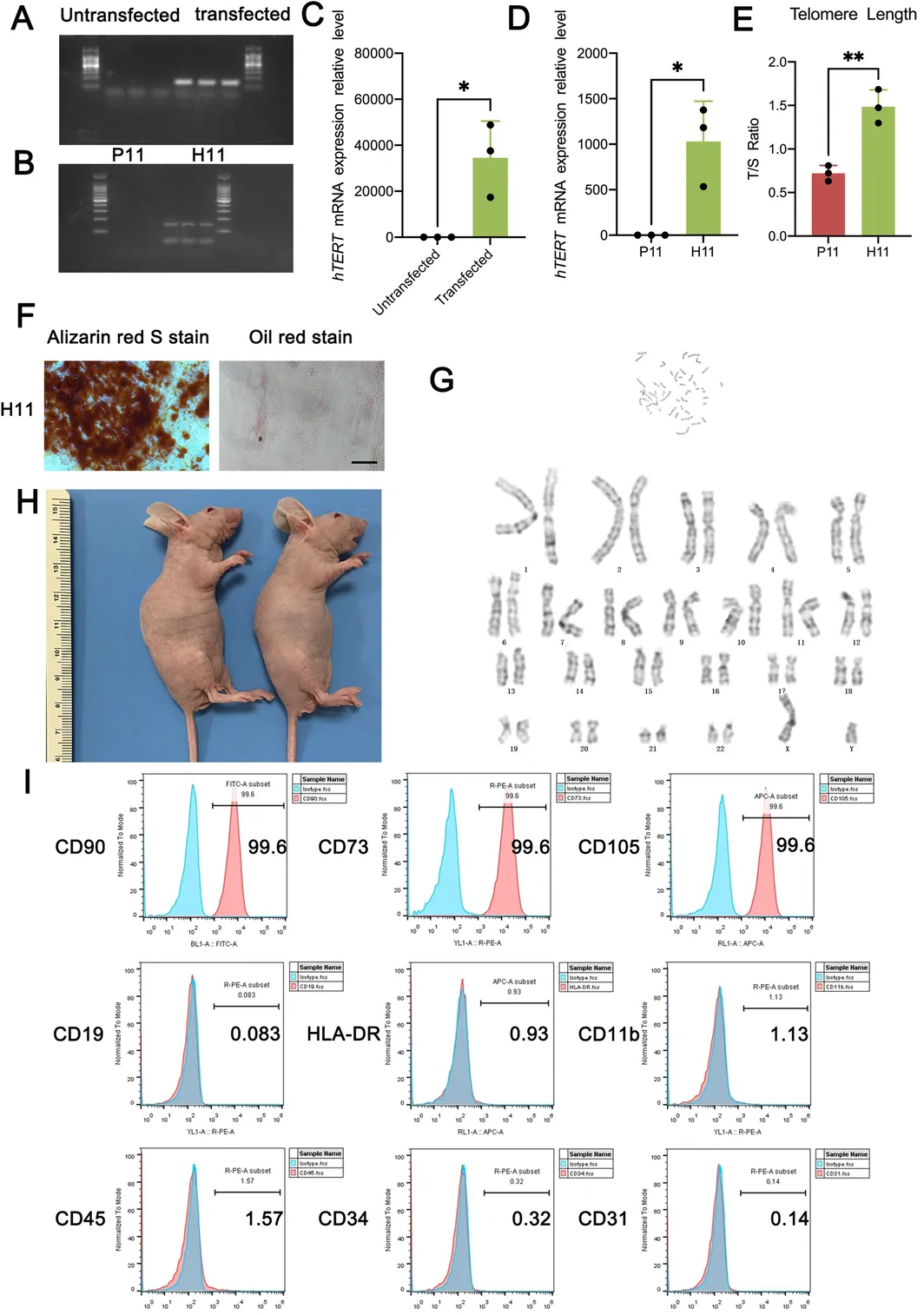
Establishment and characterization of hTERT‐immortalized hUCMSCs. (A) Verification of successful hTERT transduction in passage‐4 (P4) hUCMSCs by RT‐PCR. (B) Confirmation of hTERT expression in hTERT‐UCMSCs at passage 11 (P11) by RT‐PCR. (C), (D) mRNA expression levels of hTERT in P4 and P11 cells detected by RT‐qPCR, with GAPDH used as the internal reference gene. (E) Telomere length analysis based on the T/S ratio, representing the relative telomere (T) content to single‐copy gene (S) content. (F) Alizarin Red staining of mineralized matrix after 21 days of osteogenic induction and Oil Red O staining of lipid accumulation after 14 days of adipogenic induction in hTERT‐MSCs. (G) Karyotype analysis of hTERT‐UCMSCs. (H) Tumor‐bearing assay of hTERT‐UCMSCs. (I) Flow cytometric analysis of MSC‐associated surface markers (CD73, CD90, CD105) and negative markers (CD45, CD34, CD31, CD19, HLA‐DR, CD11b) in hTERT‐UCMSCs at passage 11. Data are expressed as mean ± SEM. *p < 0.05, **p < 0.01. Scale bar = 20 μm.

**FIGURE 3:**
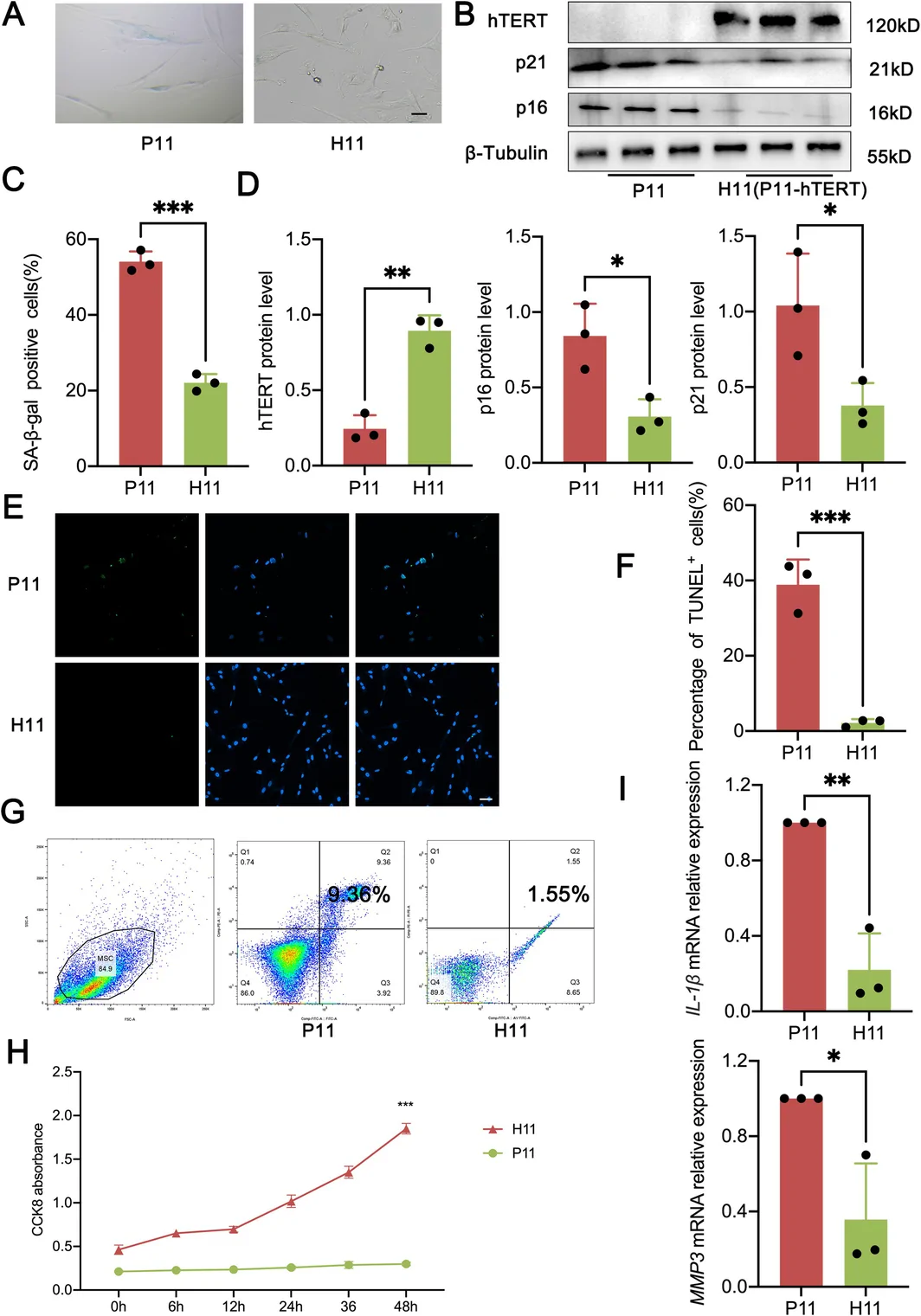
Senescence‐associated characteristics of hTERT‐UCMSCs. (A) β‐galactosidase staining of P11‐UCMSCs and H11‐UCMSCs. (B) Western blot analysis of hTERT and the senescence‐associated factors p16 and p21 in P11‐UCMSCs and H11‐UCMSCs. (C) Quantification of β‐galactosidase‐positive cells. (D) Densitometric analysis of protein expression levels by ImageJ grayscale scanning. (E) TUNEL staining of each group. (F) Quantification of TUNEL‐positive cells. (G) Annexin V–based flow cytometry assay of apoptosis. (H) CCK‐8 assay of P11 and H11 cells. (I) RT‐qPCR analysis of transcriptional levels of SASP‐related genes. Data are expressed as mean ± SEM. *p < 0.05, **p < 0.01, ***p < 0.001. Scale bars = 20 μm.

To generate a stable immortalized cell line (hTERT‐UCMSCs), hUCMSCs were transduced with a lentiviral vector encoding hTERT. RT‐PCR confirmed successful transduction, showing robust hTERT expression in the transduced group, whereas expression remained low in non‐transduced controls (Figure 2A–C). This elevation at the mRNA level was accompanied by a corresponding increase in hTERT protein (Figure 3B,D). At passage 11, hTERT‐UCMSCs maintained markedly higher hTERT expression than primary hUCMSCs (Figure 2D). Functionally, hTERT overexpression nearly doubled the average telomere length relative to control cells (Figure 2E). Importantly, hTERT‐UCMSCs preserved the bipotent differentiation capacity of MSCs, retaining both adipogenic and osteogenic potential (Figure 2F). Safety assessments revealed no evidence of tumor formation after 3 months of implantation into immunodeficient mice (Figure 2H), and karyotype analysis demonstrated normal chromosomal structure without malignant alterations (Figure 2G). Phenotypic profiling by flow cytometry showed that hTERT‐UCMSCs maintained the characteristic MSC immunophenotype, with positive expression of CD73, CD90, and CD105, and negative or low expression of CD19, CD34, CD45, CD31, CD11b, and HLA‐DR (Figure 2I). Collectively, these results demonstrate that hTERT‐mediated immortalization effectively extends the proliferative lifespan of hUCMSCs while preserving their differentiation potential, MSC surface marker profile, and non‐tumorigenic properties.

### 2.4 hTERT Overexpression Attenuates Senescence‐Related Phenotypes in hUCMSCs

To evaluate the anti‐aging effects of hTERT overexpression, we compared hTERT‐overexpressing P11 cells (H11) with senescent control P11 cells. CCK‐8 assays showed that H11 cells displayed significantly higher proliferative capacity than P11 controls (Figure 3H). Whereas primary hUCMSCs showed a marked increase in SA‐β‐gal activity with passaging, hTERT‐UCMSCs did not exhibit a comparable rise even at P11 (Figure 3A,C). Apoptosis induced by replicative senescence was also attenuated by hTERT overexpression. TUNEL staining revealed substantially fewer TUNEL‐positive cells in H11 compared with P11 (Figure 3E,F), a trend corroborated by PI‐based flow cytometry (Figure 3G). Consistent with these functional improvements, Western blot analysis showed that the senescence‐associated cell‐cycle inhibitors p16 and p21 were markedly reduced in H11 relative to P11, a finding corroborated by RT‐PCR results (Figure 3B,D, Figure S1C). Because senescent cells acquire a pro‐inflammatory senescence‐associated secretory phenotype (SASP), we further quantified SASP‐related transcripts by RT‐qPCR. hTERT overexpression significantly downregulated multiple SASP components, including inflammatory cytokines and matrix metalloproteinases (Figure 3I). Furthermore, we quantified the expression levels of γH2A.X protein, a well‐established marker for DNA damage response and cellular senescence. The results revealed that γH2A.X protein expression was upregulated in P11, while its expression was downregulated following hTERT rescue (Figure S1D,E). Thus, the enhanced proliferative capacity, reduced apoptosis and DNA damage, decreased p16/p21 expression, and suppression of SASP factors demonstrate that hTERT overexpression substantially mitigates replicative senescence in hUCMSCs.

### 2.5 Improvement of Mitochondrial Functions in hUCMSCs After Overexpression of hTERT

**FIGURE 4:**
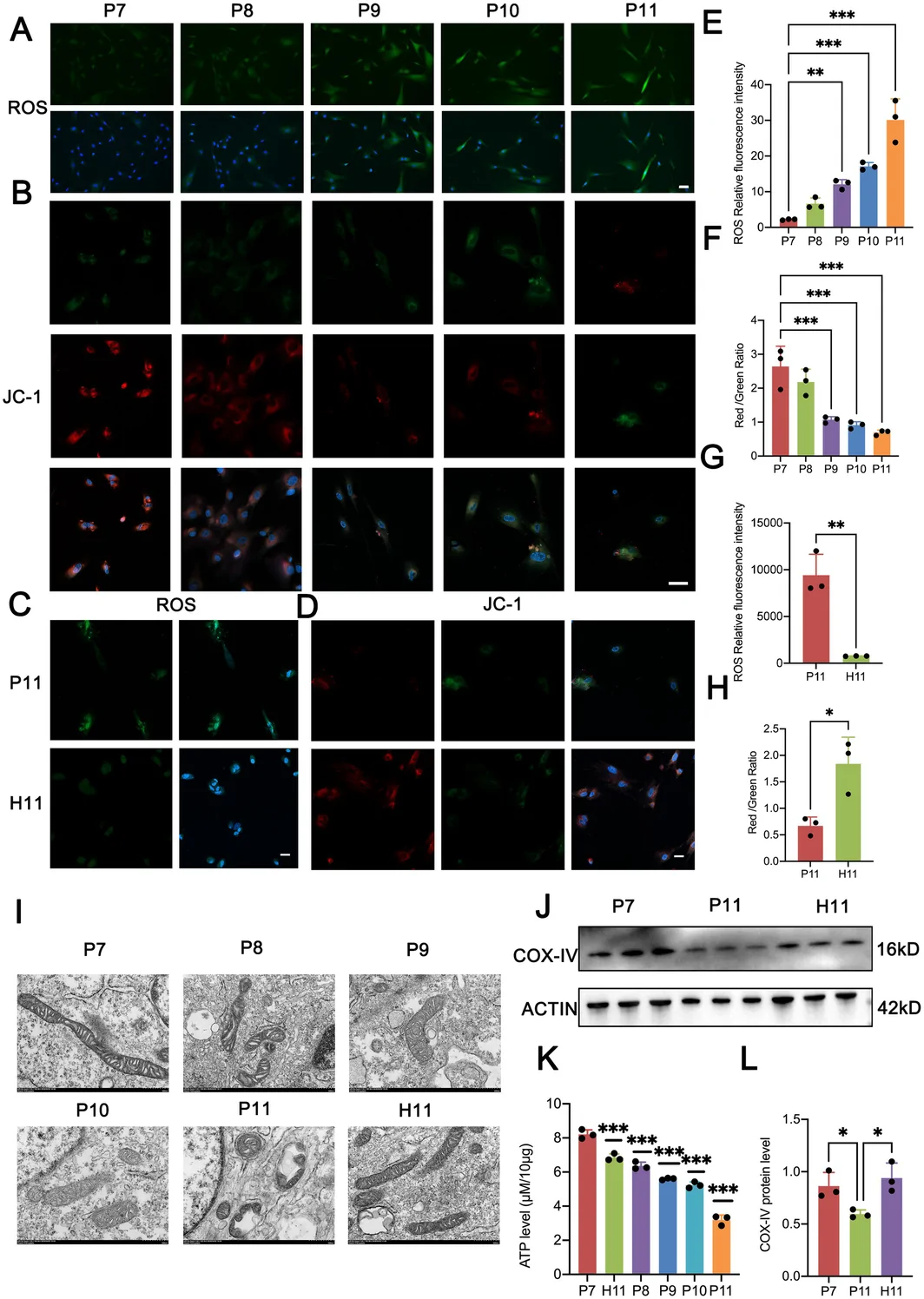
Alterations in mitochondrial function of hUCMSCs during the aging process. (A) Fluorescence images of ROS in five groups of cells, Green: ROS signal; blue: Hoechst 33342. (B) JC‐1 staining fluorescence images reflecting mitochondrial membrane potential in five groups of cells. (C) Fluorescence images of ROS levels in the P11 and H11 groups. (D) JC‐1 staining fluorescence images of mitochondrial membrane potential in the P11 and H11 groups. (E) Statistical Analysis of ROS Fluorescence Intensity in five groups of cells. (F) Statistical analysis of JC‐1 red/green fluorescence ratio across the P7–P11 groups. (G) Statistical Analysis of ROS Fluorescence Intensity in P11 and H11 groups. (H) Statistical analysis of JC‐1 red/green fluorescence ratio between the P11 and H11 groups. (I) TEM images showing mitochondrial ultrastructure in the P7–P11 and H11 groups. (J)Western blot detection of the mitochondrial marker COX‐IV protein in the P7, P11, and H11 groups. (K) Quantitative detection of intracellular ATP levels in each group. (L) Statistical analysis of relative COX‐IV protein expression levels. Data are expressed as mean ± SEM. *p < 0.05, **p < 0.01, ***p < 0.001. Scale bars = 20 μm (A–D).

Reactive oxygen species (ROS), generated as metabolic by‐products, play essential regulatory roles in MSC proliferation and differentiation under physiological conditions. Given the close relationship between mitochondrial dysfunction and ROS production, we next examined mitochondrial status during serial passaging. Using H2DCFDA staining, intracellular ROS levels were quantified in hUCMSCs from P7 to P11. Cells at P10 and P11 displayed markedly stronger green fluorescence than those at P7, indicating progressive ROS accumulation during long‐term in vitro expansion (Figure 4A,E). In P7 hUCMSCs, TOM20 immunostaining showed mitochondria were evenly distributed and predominantly exhibited elongated rod‐like morphology. In contrast, P11 cells showed disrupted mitochondrial networks with uneven distribution and a substantial shift toward punctate and fragmented mitochondria (Figure S2A). Consistently, JC‐1 staining revealed a significant decline in mitochondrial membrane potential (ΔΨm) from P8 to P11, further supporting the loss of mitochondrial integrity with replicative aging (Figure 4B,F). To determine whether hTERT overexpression mitigates these mitochondrial defects, we then compared senescent P11 cells with hTERT‐expressing H11 cells. H2DCFDA assays demonstrated substantially reduced ROS levels in H11 cells (Figure 4C,G). Mitochondrial morphology was also better preserved, with H11 cells displaying uniform mitochondrial distribution and a predominance of elongated structures (Figure S2A). Moreover, JC‐1 analysis showed that H11 cells maintained significantly higher ΔΨm relative to P11 cells (Figure 4D,G). To further assess whether hTERT overexpression mitigates mitochondrial dysfunction in senescent hUCMSCs, we evaluated the expression of mitochondrial respiratory chain‐related proteins, mitochondrial ultrastructure, and cellular ATP production. Western blot analysis revealed that the expression of COX‐IV, a representative subunit of mitochondrial respiratory chain complex IV, was downregulated in P11 compared to P7, whereas COX‐IV expression was restored in H11 (Figure 4J,L). Consistently, transmission electron microscopy (TEM) showed that mitochondria in P7 exhibited relatively intact morphology with well‐organized cristae, while mitochondria in late‐passage senescent cells displayed structural abnormalities, including swelling, vacuolization, and cristae disruption. In contrast, H11 showed improved mitochondrial ultrastructure, with more preserved mitochondrial morphology and cristae organization (Figure 4I). Moreover, intracellular ATP levels progressively declined with increasing passage number, indicating impaired mitochondrial energy‐generating capacity in senescent hUCMSCs (Figure 4K). Collectively, these findings demonstrate that hUCMSCs undergo mitochondrial structural and functional impairment during senescence, and hTERT overexpression partially restores mitochondrial integrity and bioenergetic function.

### 2.6 Differential Regulation of Inflammation by sEVs Collected From Senescent hUCMSCs and hTERT‐UCMSCs

**FIGURE 5:**
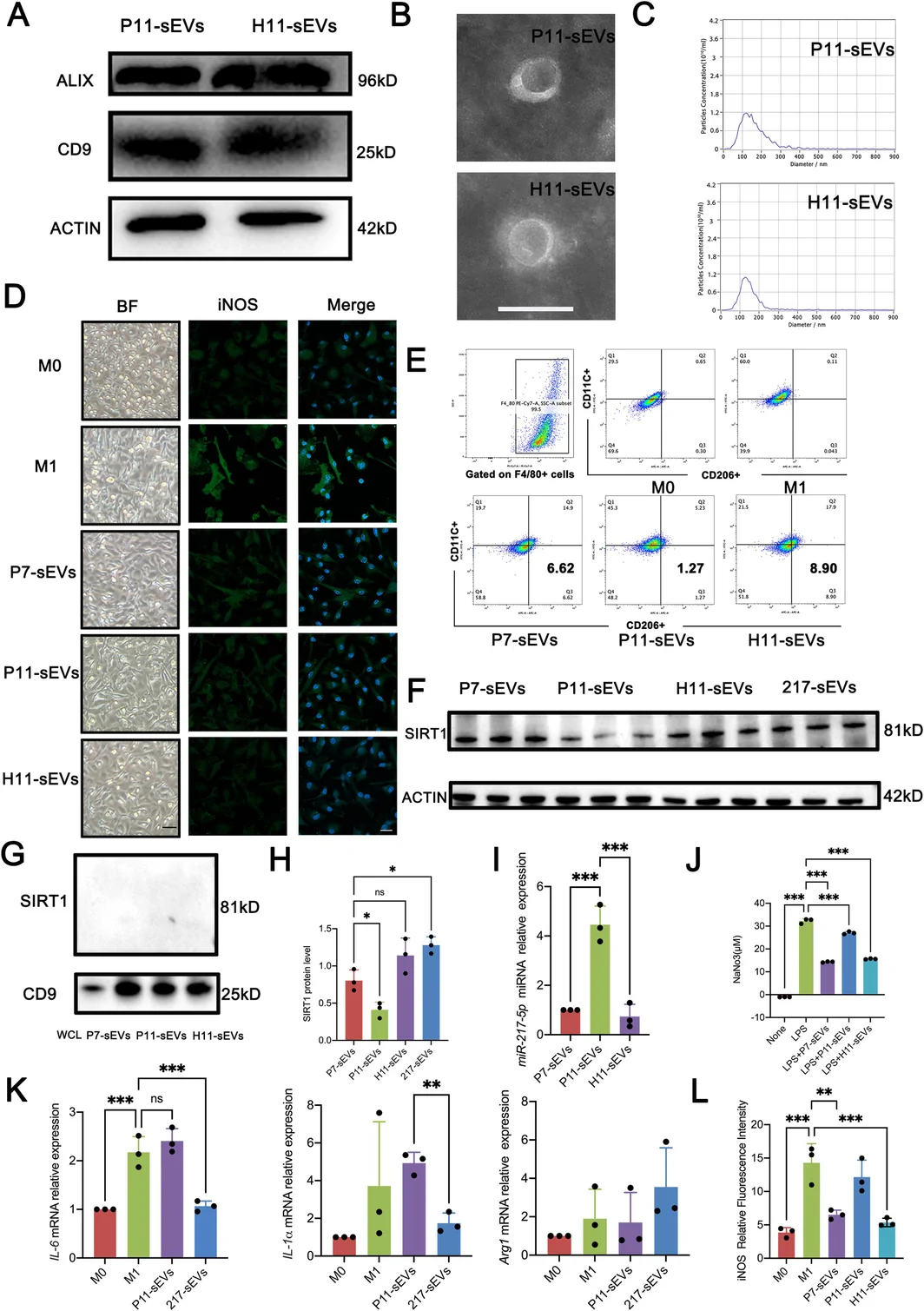
Effects of sEVs derived from P11‐ and H11‐hUCMSCs on macrophage polarization. (A) Western blot detection of characteristic sEV proteins CD9 and ALIX in P11‐ and H11‐sEVs. (B) Transmission electron microscopy images showing the morphology of sEVs from both groups, displaying typical double‐membrane circular structures. (C) Nanoparticle tracking analysis (NTA) of sEVs, showing particle size distribution and concentration. (D‐E) Mouse bone marrow‐derived macrophages (BMDMs) were polarized to the M1 phenotype by 24‐h LPS treatment, followed by incubation with P7‐, P11‐, or H11‐sEVs (1 × 10^10^ particles/mL) for an additional 24 h. Morphological changes, iNOS immunofluorescence (green; nuclei in blue), and flow cytometry were used to assess M1/M2 polarization. (F) Western blot analysis was performed to examine the changes in SIRT1 protein expression in RAW264.7 cells following treatment with P7‐sEVs, P11‐sEVs, H11‐sEVs, and 217‐sEVs. 217‐sEVs refer to P11‐sEVs that have been treated with antagomir‐217‐5p (G) Western blot detection of SIRT1 and sEVs marker CD9 in WCL, P7‐sEVs, P11‐sEVs, and H11‐sEVs. (H) Statistical analysis of relative SIRT1 protein expression levels. (I) Expression levels of miR‐217‐5p in P7‐, P11‐, and H11‐sEVs. (J) Quantitative analysis of NO production in LPS‐stimulated BMDMs treated with different sEVs groups. (K) After LPS‐induced M1 polarization, BMDMs were treated with P11‐sEVs or sEVs containing Antagomir‐217‐5p (1 × 10^10^ particles/mL) for 24 h, and the mRNA levels of pro‐ and anti‐inflammatory genes were measured. (L) Quantification of iNOS fluorescence intensity in BMDMs from each group. Scale bars = 200 nm (B) and 20 μm (D).

We next evaluate the functional differences between P11 hUCMSCs and H11 hUCMSCs by collecting sEVs from the conditional media. Their identity and purity were verified using Western blotting, transmission electron microscopy (TEM), and nanoparticle tracking analysis (NTA). Western blot analysis showed robust enrichment of the canonical exosomal markers Alix and CD9 in both preparations (Figure 5A). TEM imaging confirmed the expected cup‐shaped vesicular morphology with an average diameter of ~100 nm (Figure 5B). NTA revealed comparable particle size distributions, with P11‐sEVs measuring 82.2–145.1 nm and H11‐sEVs measuring 108.5–130.8 nm (Figure 5C). These findings indicate that sEVs released by senescent and hTERT‐restored hUCMSCs exhibit similar biophysical characteristics.

Given the established role of MSC‐derived sEVs in modulating inflammation, we next examined whether sEVs from different cellular states differentially regulated macrophage polarization. Primary bone marrow–derived macrophages (BMDMs) were cultured with P7‐sEVs (young), P11‐sEVs (senescent), or H11‐sEVs (hTERT‐overexpressing) and stimulated with LPS for 48 h. LPS induced the typical transition from M0 to M1 morphology, characterized by enlarged cell bodies and elongated projections (Figure 5D). Relative to P11‐sEVs, H11‐sEVs markedly attenuated the M1 phenotype. Immunofluorescence staining demonstrated reduced iNOS expression in the H11‐sEV group (Figure 5D,L). Flow cytometry further confirmed that both P7‐sEVs and H11‐sEVs significantly increased the proportion of F4/80^+^CD206^+^CD11c^−^ macrophages, indicating enhanced M2 polarization, whereas P11‐sEVs exhibited a diminished capacity to promote this phenotype (Figure 5E). To further validate the effects of different sEVs on macrophage inflammatory activation, we supplemented NO production assays in LPS‐stimulated BMDMs treated with P7‐sEVs, P11‐sEVs, or H11‐sEVs (Figure 5J). The results showed that P11‐sEVs exhibited a weaker inhibitory effect on NO production compared with P7‐sEVs, whereas H11‐sEVs restored the ability to suppress NO production. We also conducted qRT‐PCR analysis on representative M1 and M2 macrophage markers (Figure S2B). Compared with P11‐sEVs, H11‐sEVs more effectively downregulated the expression of M1‐associated markers and upregulated the expression of M2‐related genes, including ARG1 and IL‐10. Together with the results from immunofluorescence and flow cytometry, these data further demonstrate that senescence impairs the anti‐inflammatory activity of hUCMSC‐derived sEVs, whereas hTERT overexpression helps preserve their inhibitory effect on macrophage inflammatory responses. To explore whether senescence‐associated miRNAs contribute to the impaired immunomodulatory activity of P11‐sEVs, we screened several candidate miRNAs previously linked to cellular senescence and inflammatory responses (Menghini et al. 2009; Wallis et al. 2020; Yamakuchi 2012). Among these candidates, miR‐217‐5p showed the most obvious enrichment in P11‐sEVs compared with P7‐sEVs (Figure 5I). Additionally, prior studies have reported that miR‐217 is enriched in senescence‐associated sEVs and propagates pro‐senescent and pro‐inflammatory signals (Mensà et al. 2020; Papageorgiou et al. 2023; Zhang et al. 2024). Therefore, miR‐217‐5p was selected for subsequent functional validation. We next compared miR‐217‐5p expression between young and senescent sEVs. qRT‐PCR showed that miR‐217‐5p was significantly upregulated in P11‐sEVs compared with P7‐sEVs, whereas its expression in H11‐sEVs resembled that of young sEVs (Figure 5I). To further investigate the potential downstream mechanism linking miR‐217‐5p enrichment to the impaired immunomodulatory activity of senescent sEVs, studies have shown that miR‐217 can regulate SIRT1 (Yamakuchi 2012), we assessed SIRT1 expression in donor hUCMSCs, recipient macrophages, and sEV cargo. First, SIRT1 expression was reduced in P11 compared with P7, whereas H11 showed restored or maintained SIRT1 expression (Figure S1D,E). These findings are consistent with the established role of SIRT1 in maintaining cellular homeostasis and counteracting senescence‐associated alterations. We also evaluated whether SIRT1 itself was directly carried by hUCMSC‐derived sEVs. SIRT1 protein was not detected in sEV cargo (Figure 5G). This suggests that the regulation of macrophage SIRT1 expression by senescent sEVs is unlikely to result from direct transfer of SIRT1, but is more likely mediated by senescence‐associated miRNA cargo, particularly miR‐217‐5p. To determine whether reducing miR‐217‐5p could restore the immunomodulatory function of senescent sEVs, P11‐sEVs were treated with antagomir‐217‐5p before being applied to LPS‐stimulated BMDMs. After 48 h, qRT‐PCR analysis demonstrated that inhibition of miR‐217‐5p significantly downregulated the expression of IL‐6 and IL‐1α, while upregulating the expression of Arg1 (Figure 5K). We next examined whether antago‐miR‐217‐5p loading affected the basic properties of sEVs. NTA showed that treated sEVs displayed particle‐size distribution and concentration comparable to untreated sEVs. Western blotting confirmed that treated sEVs retained CD9, CD63, and HGS expression, while Calnexin was undetectable (Figure S2C,D). These data suggest that antago‐miR‐217‐5p treatment did not markedly alter the basic characteristics of sEVs. We next examined whether sEVs derived from different cellular states affected SIRT1 expression in recipient macrophages (Figure 5F,H). Compared with P7‐sEVs, P11‐sEVs reduced SIRT1 expression in LPS‐stimulated RAW264.7 cells and exhibited weakened anti‐inflammatory activity. In contrast, H11‐sEVs better preserved SIRT1 expression in recipient macrophages, consistent with their stronger ability to suppress M1 polarization and promote an M2‐like phenotype. Furthermore, inhibition of miR‐217‐5p in P11‐sEVs restored SIRT1 expression in recipient macrophages, accompanied by reduced expression of pro‐inflammatory cytokines and increased expression of anti‐inflammatory markers (Figure 5K).

These data suggest that miR‐217‐5p contributes to the impaired anti‐inflammatory activity of senescent sEVs and that its downregulation partially restores their ability to promote M2 polarization. Together, these findings support a model in which senescence‐associated enrichment of miR‐217‐5p in hUCMSC‐derived sEVs suppresses SIRT1 expression in recipient macrophages, thereby weakening the immunomodulatory potency of senescent sEV. Conversely, hTERT rescue is associated with reduced senescence‐related miR‐217‐5p enrichment in sEVs, preservation of SIRT1‐associated anti‐inflammatory signaling in macrophages, and maintenance of the immunomodulatory activity of hUCMSC‐derived sEVs.

### 2.7 Comparable Effects of Senescent and hTERT‐Overexpressing hUCMSC‐Derived sEVs on Primordial Follicle Activation and Ovarian Protective Activities

**FIGURE 6:**
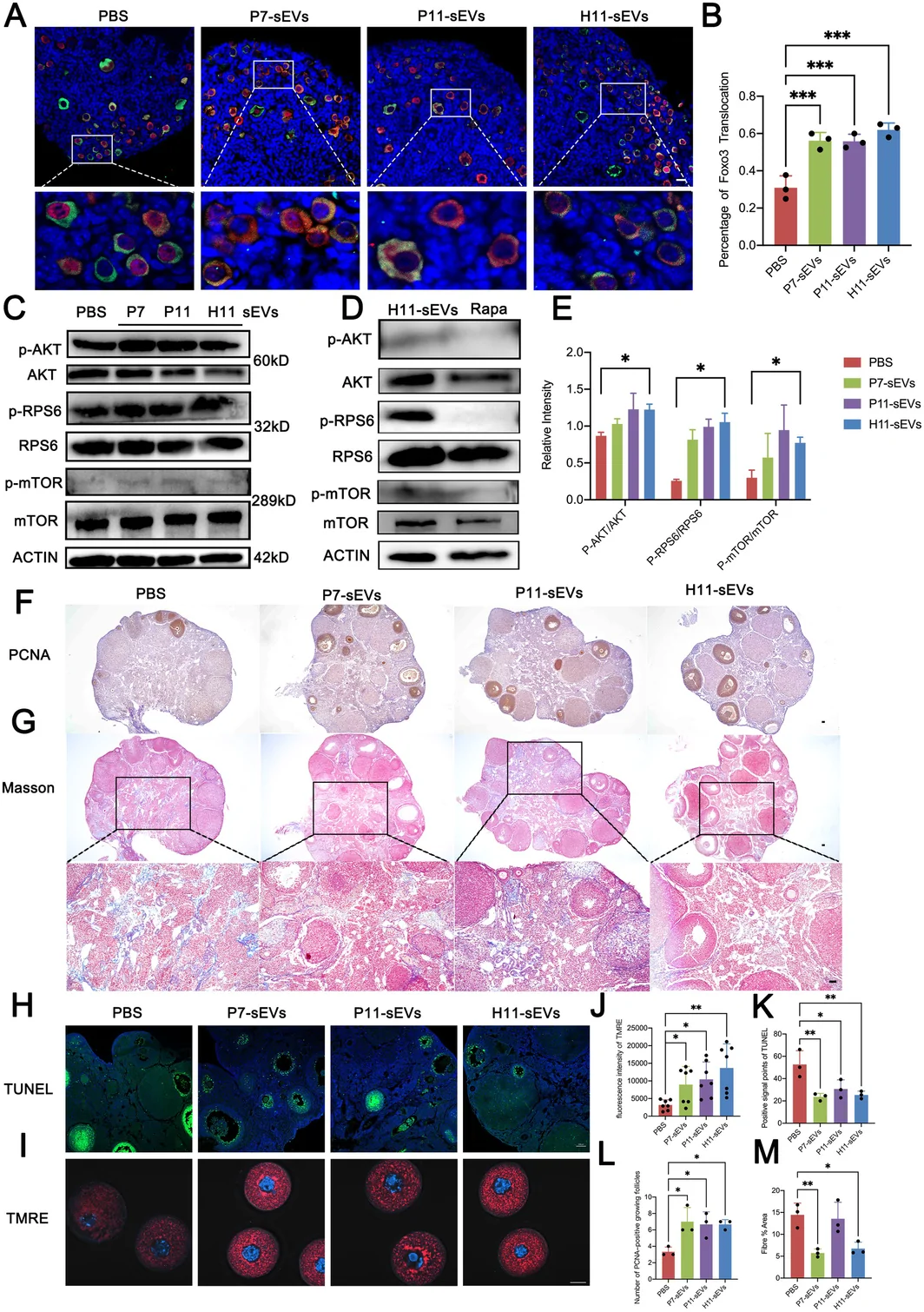
Comparison of the effects of P11‐sEVs and H11‐sEVs on primordial follicle activation and ovarian protective activities. (A) Mouse ovaries from 2.5‐day‐old pups were cultured in vitro for 48 h with P7‐, P11‐, or H11‐sEVs (1 × 10^10^ particles/mL), followed by immunofluorescence staining for DDX4 (green) and FOXO3a (red). PBS was used as a control. The large panel shows a magnified view of the region indicated by the small white box, representing activated or dormant primordial follicles. (B) Quantification of the proportion of FOXO3a exiting the nucleus. (C) Expression of the PI3K‐AKT–mTOR signaling pathway proteins in ovaries treated with P11‐ or H11‐sEVs; P7‐sEVs served as a positive control. (D) Additional co‐treatment with the mTOR inhibitor Rapamycin (Rapa) and sEVs was performed, followed by analysis of protein expression in the pathway. (E) Ratios of phosphorylated proteins to their respective total protein levels as measured in panel (C). (F) PCNA immunohistochemical staining of ovarian sections. (G) Masson's trichrome staining of ovarian tissue sections, with enlarged views of interstitial fibrosis regions indicated by black boxes. (H) TUNEL fluorescence staining detecting apoptotic signals in ovarian oocytes; green: TUNEL, blue: Hoechst. (I) TMRE staining for detecting oocyte mitochondrial membrane potential (red fluorescence). (J) Quantification of TMRE fluorescence intensity. (K) Quantification of TUNEL fluorescence intensity, Green: TUNEL signal; blue: Hoechst 33342. (L) Quantitative analysis of PCNA‐positive growing follicles in the largest cross‐section of the ovary. (M) Quantification of ovarian fibrosis by Masson‐stain. Data are expressed as mean ± SEM. *p < 0.05, **p < 0.01, ***p < 0.001. Scale bars = 50 μm (A, F–G), 20 μm (I), and 100 μm (H).

In our previous work, we demonstrated that MSC‐derived sEVs can promote primordial follicle activation (Yang et al. 2020). To further determine whether functional differences exist between P11‐sEVs and H11‐sEVs, we employed an established neonatal ovarian culture model. This assay evaluates the capacity of sEVs to stimulate oocyte activation and early follicular growth. Neonatal ovaries were incubated for 48 h with P7, P11, or H11‐sEVs. Activation of primordial follicles was assessed by FOXO3a subcellular localization, as FOXO3a translocation from the oocyte nucleus to cytoplasm is a hallmark of primordial follicle activation. Immunofluorescence staining for DDX4 and FOXO3a showed comparable proportions of activated primordial follicles across all sEVs‐treated groups (Figure 6A,B), indicating that young, senescent, and hTERT‐restored sEVs each retain the ability to trigger follicle activation. Since activation of the PI3K and mTOR pathways is essential for primordial follicle awakening, we next examined pathway activation by Western blotting. Treatment with P7‐sEVs and H11‐sEVs resulted in increased phosphorylation of Akt, mTOR, and ribosomal protein S6, confirming activation of both the PI3K and mTOR signaling pathways. Interestingly, P11‐sEVs also induced similar increases in p‐Akt, p‐mTOR, and p‐rpS6, and no significant differences were observed among the three sEV groups (Figure 6C,E), further supporting that senescent‐derived sEVs nonetheless preserve follicle‐activating capacity. To verify pathway specificity, neonatal ovaries were treated with H11‐sEVs in the presence of the mTOR inhibitor rapamycin. Rapamycin effectively abolished sEV‐induced activation of both the PI3K and mTOR pathways (Figure 6D), confirming that PI3K/mTOR signaling is required for H11‐sEVs‐mediated follicle activation. Together, these findings demonstrate that sEVs derived from young, senescent, and hTERT‐overexpressing hUCMSCs similarly promote primordial follicle activation through the PI3K/mTOR signaling axis, despite differences in their parental cell states. To evaluate the in vivo activity of hUCMSC‐derived sEVs, 10‐month‐old female ICR mice were treated with PBS, P7‐sEVs, P11‐sEVs, or H11‐sEVs by tail vein injection once weekly for 4 weeks. Each sEVs‐treated mouse received 1.0 × 10^10^ particles per injection. Compared to PBS‐treated mice, all sEVs‐treated groups showed an increased number of PCNA‐positive mature follicles, indicating enhanced proliferation capacity of granulosa cells. Compared to PBS‐treated mice, all sEVs‐treated groups showed an increased number of PCNA‐positive growing follicles, indicating enhanced follicular cell proliferation (Figure 6F,L). Masson staining revealed obvious fibrosis in the ovaries of both the PBS and P11‐sEVs groups, whereas P7‐sEVs and H11‐sEVs treatments reduced collagen deposition (Figure 6G,M). TUNEL staining indicated decreased apoptotic signals following treatment with the three types of sEVs (Figure 6H,K). Additionally, TMRE staining of GV‐stage oocytes demonstrated improved mitochondrial membrane potential in all three sEVs‐treated groups (Figure 6I,J). Together, these in vivo results indicate that all three sEVs exert beneficial effects on the aged ovarian microenvironment. Notably, H11‐sEVs showed effects comparable to young P7‐sEVs, whereas senescent P11‐sEVs displayed relatively weaker activity, particularly in reducing ovarian fibrosis. These findings support the conclusion that hTERT‐rescued hUCMSCs produce sEVs with preserved ovarian protective and follicle‐supporting activity in vivo.

### 2.8 hTERT Preserves Young‐Like sEV Protein Profiles

**FIGURE 7:**
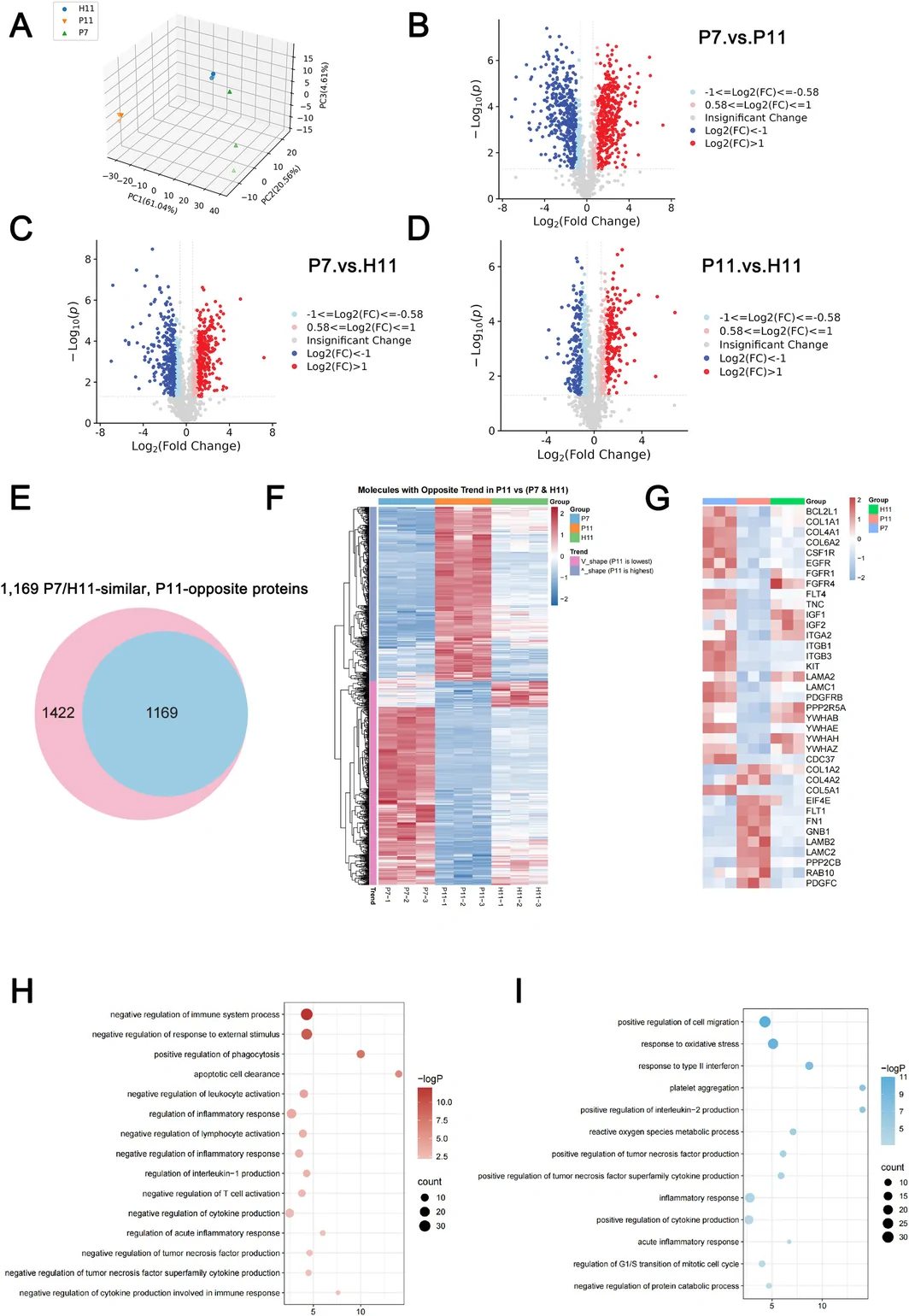
Proteomic profiling of sEVs derived from P7, P11, and H11 hUCMSCs. (A) 3D PCA plot showing the clustering of sEV proteome samples from P7, P11, and H11 groups.(B–D) Volcano plots displaying differentially expressed proteins for pairwise comparisons: P7‐sEVs vs. P11‐sEVs, P7‐sEVs vs. H11‐sEVs, and P11‐sEVs vs. H11‐sEVs.(E‐F) Venn and heatmap diagrams illustrating differentially expressed proteins (DEPs) from P7‐sEVs, P11‐sEVs, and H11‐sEVs.(G) Heatmap of proteins associated with the PI3K‐Akt signaling pathway in P7‐sEVs, P11‐sEVs, and H11‐sEVs. (H) Bubble plot of enriched GO biological processes for downregulated proteins in P11‐sEVs. (I) Bubble plot of enriched GO biological processes for upregulated proteins in P11‐sEVs.

To further investigate whether cellular senescence and hTERT overexpression alter the protein cargo of sEVs derived from hUCMSCs, we performed proteomic profiling of P7‐sEVs, P11‐sEVs, and H11‐sEVs. Principal component analysis (PCA) revealed a clear separation among the three groups, indicating distinct sEV protein profiles associated with young, senescent, and hTERT‐rescued cellular states (Figure 7A). Volcano plot analysis further identified numerous differentially expressed proteins (DEPs) in pairwise comparisons between the groups, suggesting that senescence markedly reshapes the protein composition of hUCMSC‐derived sEVs (Figure 7B–D). Venn diagram analysis identified 1422 DEPs across P7‐sEVs, P11‐sEVs, and H11‐sEVs. Notably, 1169 of these proteins exhibited similar expression trends in P7‐sEVs and H11‐sEVs but displayed opposite trends in P11‐sEVs (Figure 7E). We therefore focused on this subset for subsequent analyses, as these proteins may represent young‐like sEV cargo that is lost or dysregulated during senescence but preserved or restored by hTERT overexpression. Heatmap analysis further confirmed that this subset of proteins showed opposite expression patterns in P11‐sEVs compared to P7‐sEVs and H11‐sEVs, supporting the notion that hTERT overexpression preserves a young‐like sEV protein signature (Figure 5F). Given the critical role of the PI3K‐Akt signaling pathway in primordial follicle activation, we further examined PI3K‐Akt pathway‐related proteins in the sEV proteomic profiles. A panel of PI3K‐Akt pathway‐associated proteins was detected across P7‐sEVs, P11‐sEVs, and H11‐sEVs, including growth factor receptors, extracellular matrix‐related proteins, integrins, and downstream signaling‐associated proteins. Although individual proteins showed group‐dependent variations in abundance, the overall PI3K‐Akt pathway‐related protein signature was broadly retained across all three sEV groups (Figure 7G). These results suggest that P7‐sEVs, P11‐sEVs, and H11‐sEVs may all possess the potential to support PI3K‐Akt‐related signaling activity, consistent with their shared capacity to promote primordial follicle activation.

Functional enrichment analysis of the same protein subset revealed that proteins downregulated in P11‐sEVs but maintained or restored in P7‐sEVs and H11‐sEVs were enriched in biological processes related to negative regulation of immune system processes, negative regulation of leukocyte and lymphocyte activation, negative regulation of cytokine production, negative regulation of TNF production, positive regulation of phagocytosis, and apoptotic cell clearance (Figure 7H). These biological processes are closely associated with the suppression of inflammatory activation and resolution of inflammation, indicating that senescence leads to the loss of anti‐inflammatory and immunoregulatory protein signatures in hUCMSC‐derived sEVs.

Conversely, proteins upregulated in P11‐sEVs were enriched in pathways related to oxidative stress response, IL‐2 and TNF production, reactive oxygen species metabolic process, inflammatory response, cytokine production, and cell‐cycle regulation (Figure 7I). These results suggest that senescence shifts the sEV protein cargo toward a more pro‐inflammatory and stress‐associated profile. Collectively, the proteomic data indicate that all hUCMSC‐derived sEV groups retain PI3K‐Akt pathway‐related protein features associated with follicle activation, while hTERT overexpression further helps preserve a young‐like immunomodulatory protein cargo, thereby providing a molecular basis for the retained follicle‐activating and anti‐inflammatory activities of H11‐sEVs.

## 3 Discussion

The clinical translation of MSC‐based therapies, particularly those leveraging paracrine effects via secretomes and small extracellular vesicles (sEVs), has long been constrained by inherent biological limitations, including cellular senescence and heterogeneity (Chouaib et al. 2023; Kou et al. 2022). In this study, we explored the potential of hTERT‐overexpressing hUCMSCs (hTERT‐UCMSCs) as a standardized source for sEV production. Notably, sEVs derived from hTERT‐UCMSCs exhibited particle yields comparable to those from low‐passage control MSCs, effectively addressing the challenge of production scale. More importantly, these sEVs retained robust biological activity, closely resembling that of young MSC‐derived sEVs, and in some aspects, surpassed the functionality of sEVs from senescent cells. Functional assays demonstrated that hTERT‐UCMSC sEVs effectively promoted M2 macrophage polarization and activated primordial follicles, highlighting their dual immunomodulatory and regenerative potential. These findings underscore the possibility of harnessing sEVs from immortalized MSCs for therapeutic applications, including reproductive medicine and immune‐mediated disorders, such as chronic inflammation, ischemia–reperfusion injury, and autoimmune diseases.

A primary challenge in MSC‐based therapies lies in the limited proliferative capacity of primary MSCs due to progressive telomere shortening, ultimately leading to growth arrest (the Hayflick limit) (Shay and Wright 2000; Wagner et al. 2008). This not only restricts the yield of therapeutic cells but also contributes to the acquisition of a senescence‐associated secretory phenotype (SASP) (Weng et al. 2022), compromising their functional integrity. Our results demonstrate that hTERT overexpression effectively overcomes this limitation by extending telomere length and preserving proliferation. Crucially, this immortalization process did not induce tumorigenicity, as evidenced by stable karyotypes and the absence of tumor formation in vivo, while maintaining normal mesenchymal stem cell surface markers and multipotent differentiation capacity. These features are critical for ensuring the safety and efficacy of MSC‐based regenerative therapies.

Mechanistically, we identified miR‐217‐5p as an important candidate cargo of sEVs that mediates their influence on macrophage polarization. Senescent MSC‐sEVs exhibited significantly higher levels of miR‐217‐5p compared with young or hTERT‐UCMSC‐derived sEVs, correlating with their diminished capacity to induce M2 polarization. Functional inhibition of miR‐217‐5p in senescent sEVs restored anti‐inflammatory effects, suggesting that miRNA cargo is a central determinant of sEV function. Notably, although antago‐miR‐217‐5p treatment provided functional evidence for the involvement of miR‐217‐5p, direct manipulation of isolated sEVs has methodological limitations. Future studies using parental‐cell miR‐217‐5p knockdown or knockout will be needed to further confirm this mechanism. Previous studies have shown that miR‐217 modulates SIRT1 expression, thereby influencing both cellular senescence and inflammatory responses (Khor and Wong 2025; Zhang et al. 2024), which is consistent with our findings. However, the therapeutic activity of MSC‐sEVs is likely the result of a complex interplay between multiple molecular components (Tian et al. 2025). Other miRNAs, such as miR‐30b‐5p (Xiao et al. 2022), as well as proteins and lipids enriched in senescent sEVs, can modulate NF‐κB signaling and inflammatory pathways (Patel et al. 2025; Zhang et al. 2024). This emphasizes the need for comprehensive profiling of sEV cargo to fully elucidate their mechanisms of action.

Despite these promising results, several areas warrant further investigation. Long‐term monitoring of genomic stability in hTERT‐UCMSCs at higher passage numbers is necessary to exclude late‐onset genomic alterations. Comparing with very early passages such as P2 or P3 will provide a broader aging lineage. In the present study, proteomic profiling has provided additional molecular evidence that H11‐sEVs retain young‐like immunomodulatory protein features; however, integration with miRNA and lipidomic datasets will be needed to define the complete functional cargo network of hTERT‐UCMSC‐derived sEVs. Finally, preclinical evaluation of hTERT‐UCMSC sEVs in complex in vivo disease models that closely mimic human pathophysiology is essential to validate their therapeutic efficacy and safety. Collectively, our study establishes hTERT‐UCMSCs as a promising platform for the standardized production of biologically active sEVs and provides mechanistic insights into their regenerative and immunomodulatory potential.

## 4 Materials and Methods

### 4.1 Mice

All mice used in this study were purchased from Nanjing Medical University and raised in the Animal Research Center of Nanjing Medical University (Nanjing, China). Animals were kept under standardized controlled housing conditions: a 12 h light/dark cycle, constant temperature of 22°C, with free access to standard chow and sterile drinking water. The entire animal experimental protocol was reviewed and approved by the Animal Ethics Committee of Nanjing Medical University (Approval No.: IACUC‐2211065 & IACUC‐2603081), and all animal operations strictly complied with the ARRIVE guidelines 2.0.

Two separate animal experiments were carried out in this work: a tumorigenicity safety test using athymic nude mice, and an in vivo therapeutic experiment using aged female ICR mice.

#### 4.1.1 Tumorigenicity Safety Experiment

Two 4‐week‐old athymic nude mice were utilized for safety detection. Briefly, 1 × 10^6^ hTERT‐overexpressing hUCMSCs were resuspended in 100 μL sterile PBS and subcutaneously injected into the right rib area of each nude mouse. Three months post‐injection, all nude mice were humanely sacrificed via inhalation of an overdose of carbon dioxide for subsequent tumorigenesis inspection.

#### 4.1.2 In Vivo Therapeutic Experiment With Aged ICR Mice

A total of 24 female ICR mice at 10 months of age were included and randomly assigned to four groups (*n* = 6 per group): PBS control group, P7 hUCMSC‐sEVs treatment group, P11 hUCMSC‐sEVs treatment group, and H11 hTERT‐overexpressing hUCMSC‐sEVs treatment group. Every mouse was marked with an ear tag, and group randomization was generated by computer; each individual mouse was regarded as one independent experimental unit.

For intervention administration, tail vein intravenous injection was performed once a week for four consecutive weeks. For the three sEV treatment groups, each mouse received 1.0 × 10^10^ sEV particles diluted in a unified volume of PBS. Mice in the PBS control group were injected with the same volume of sterile PBS following the identical injection frequency and route.

All housing, injection, and sampling operations were completed in the Animal Research Center of Nanjing Medical University. The researcher who performed injections and sample processing was blinded to group allocation and only obtained grouping information after all treatments ended. An independent biostatistician completed outcome evaluation and statistical analysis, and at least two researchers cross‐checked all histological and cellular detection results to avoid observer bias.

Three days after the final tail vein injection, all aged ICR mice were humanely euthanized by cervical dislocation. Bilateral ovarian tissues were quickly isolated and collected for subsequent histological assessment and oocyte quality analysis.

### 4.2 Isolation and Culture of hUCMSCs

Fresh umbilical cords were collected from parturients who provided informed consent at Sir Run Run Hospital, Nanjing Medical University. This study was approved by the Ethics Committee of Nanjing Medical University (Approval No.: (2018) 371). The isolation of hUCMSCs was performed according to a previously described method. The phenotype of P11 and H11 were characterized by flow cytometry, using antibodies specific for positive markers CD73, CD90, and CD105, as well as antibodies against negative markers CD11b, CD19, CD14, CD34, CD45, and HLA‐DR. During passages 7–11, the differentiation potential of hUCMSCs was evaluated using osteogenic medium (Product No. FY200006, Fuyuan Biotechnology Co. Ltd., China) and adipogenic medium (Product No. FY200007, Fuyuan Biotechnology Co. Ltd., China). Differentiated cells were identified by alizarin red staining for osteogenic differentiation and oil red O staining for adipogenic differentiation, respectively.

### 4.3 Isolation of sEVs by Tangential Flow System and Size‐Exclusion Chromatography

The isolation of hUCMSC‐sEVs was carried out as previously reported (Liu et al. 2025). Briefly, the supernatant was first processed through a tangential flow filtration (TFF) system. The filtrate was collected using a MaxCell hollow‐fiber filter cartridge (750,000 NMWC, Cytiva, USA). This filtrate was then further filtered through a 300,000 NMWC hollow‐fiber filter cartridge, and the concentrate was collected with a final volume ranging from 15 to 30 mL. Subsequently, the concentrate was injected into a size‐exclusion column (Superdex 200 HiLoad 26/60, Cytiva, USA). The sEVs were separated using an ÄKTA pure chromatography system (Cytiva, USA). The column temperature was set at 6°C. With PBS buffer as the mobile phase, the flow rate was set at 0.5 mL/min, and each fraction of 4 mL was collected. This procedure was repeated until all the concentrates had been fully processed.

### 4.4 NTA Measurement Using Zetaview (Particle Metrix, Germany)

The particle size distribution and concentration of samples were analyzed by employing the ZetaView nanoparticle tracking analyzer (Model: Zetaview—PMX120—Z; Software Version: 8.05.14 SP7) manufactured by Particle Metrix GmbH, Germany.

Prior to measurement, the samples were diluted with 1X phosphate‐buffered saline (PBS) buffer to an appropriate concentration, aiming for an ideal particle count of 140–200 particles per frame. The measurements were conducted at a constant temperature of 25°C. For each sample, three measurement cycles were executed. In each cycle, 11 cell positions were scanned, and 60 high‐definition frames were captured at each position. Subsequently, the captured videos were analyzed using the ZetaView software with specific analysis parameters: Maximum particle size: 1000, Minimum particle size: 10, Minimum brightness: 30, and Maximum brightness: 255.

### 4.5 Transmission Electron Microscopy (TEM)

Approximately 30 μL of the sEVs sample was dropped onto a carbon–coated copper grid placed on a sealing film. The sample was allowed to stand for 5 min. Subsequently, a pointed filter paper was used to absorb the excess solution from the edge of the grid, and then the grid was placed on the filter paper and left to stand for approximately 10 min. After the support film had dried, a drop of uranyl acetate staining solution was added, and the sample was stained for 90 s. Then, the excess staining solution was blotted off, and the support film with the grid was clamped between filter papers and dried for 3 h prior to observation.

### 4.6 Macrophage Polarization Induction Experiment

Bone marrow was harvested from the femur and tibia using a 10–mL syringe equipped with needles. The flushed cells were subsequently cultured in RPMI 1640 medium (Bio‐sharp, China) supplemented with 10% fetal bovine serum (FBS, cat. no. 10099141, Gibco, USA), penicillin/streptomycin (100 μg/mL), and 10% (v/v) conditioned medium of L929 mouse fibroblasts for 7 days to obtain primary mouse bone–marrow–derived macrophages (BMDMs). The mouse BMDMs were seeded in six‐well plates and treated with 100 ng/mL lipopolysaccharide (LPS) for 24 h to induce their polarization into M1 macrophages. Subsequently, three types of small sEVs, namely P7‐sEVs, P11‐sEVs, and H11–sEVs, were added to the cells at a concentration of 1 × 10^10^ particles/mL, followed by an additional 24‐h treatment. Initially, changes in cell morphology were carefully observed. Subsequently, the transition of macrophage polarization states from M1 (CD11c^+^; CD206^−^) to M2 (CD11c^−^; CD206^+^) was detected via flow cytometry. Moreover, the expression levels of the M1 macrophage marker inducible nitric oxide synthase (iNOS) in different treatment groups were examined by immunofluorescence.

### 4.7 hUCMSCs Lentiviral Transfection

The transfection procedure was carried out in accordance with the instructions of the kit (#CILV02, ALSTEM Inc., USA). P3—passage cells were selected for transfection. The cells were seeded into six—well plates at a density of 1 × 10^5^ cells per well. On the following day, the concentrated recombinant lentivirus was thawed in a water bath at 37°C. Then, 10 μL of the recombinant lentivirus and 4 μL of TransPlus were added to each well. On the third day, the viral supernatant was removed, and the cells were replenished with complete cell culture medium. After 72 h, the cells were passaged and screened with puromycin at a concentration of 1 μg/mL.

### 4.8 Detection of the Relative Telomere Length of hUCMSCs

The procedures were conducted following the instructions of the relative telomere length detection kit (Yihe Biotechnology Co. Ltd., China). Cellular DNA was extracted, followed by qPCR amplification. Based on the results of quantitative PCR, the T/S ratio was calculated. The calculation method for telomere length (T/S) is as follows: First, calculate the △CT for both the standard and the sample, where △CT = CT (telomere) − CT (internal reference). Subsequently, calculate T/S = 2^−(△CT (sample)−△CT (standard))^. It should be noted that T represents the sample to be assayed, S represents the standard, and T/S represents the relative telomere length ratio between the sample to be tested and the standard.

### 4.9 G—Banding Karyotype Analysis

One day prior to cell passage, cells were harvested for karyotype analysis.

The cells were first incubated in fresh culture medium for 2–4 h. Subsequently, they were treated with colchicine solution (Product No. A600322, Sangon Biotech Co. Ltd., China) at a final concentration of 0.02 μg/mL for 1 h.

Next, the cells were washed twice with PBS. Then, they were digested with trypsin to obtain single—cell suspensions and centrifuged. The resulting cell pellet was resuspended in 5 mL of hypotonic solution (0.075 M KCl) and incubated at 37°C for 15 min. Subsequently, 1 mL of ice—cold fixative (prepared as a 3:1 mixture of methanol to glacial acetic acid) was added dropwise to the hypotonic cell suspension, and the mixture was left at room temperature for 5 min.

After centrifugation to remove the supernatant, 5 mL of ice‐cold fixative was added dropwise to the cell pellet suspension. The suspension was then incubated at room temperature for 30 min, followed by another centrifugation step. This fixation procedure was repeated three times. Finally, 0.2 mL of the fixed cell suspension was retained for slide‐dropping. After the slides were completely dried, they were stained with 1× Giemsa staining solution. After natural air‐drying of the stained slides, microscopic observation was carried out. For each karyotype analysis, at least 20 metaphase cells were counted.

### 4.10 sEVs miRNA Interference

According to the manufacturer's instructions, the miRNA antagonist Antagomir‐217‐5p was transfected into P11‐sEVs using Lipofectamine 2000 (Invitrogen, Waltham, MA, USA). Briefly, the sEVs pellet isolated from 200 mL of conditioned medium was resuspended in 100 μL of PBS containing 100 nmol of Antagomir and 2 μL of Lipofectamine 2000 and incubated at 37°C for 4 h. After washing with PBS, the Antagomir‐containing sEVs pellet was collected by ultracentrifugation at 120,000 × g for 2 h. Finally, the pellet was resuspended in 150 μL of PBS, and BMDM were treated with the same concentration of P11‐sEVs and Antagomir‐containing sEVs (1 × 10^10^/mL).

### 4.11 In Vitro Ovarian Organ Culture

Ovaries (*n* = 10 per group) were collected from P2.5 female pups and cultured on Millicell inserts (Millipore) in 24‐well culture plates (NEST, China) containing 400 μL of ovarian culture medium. The plates were placed in a 37°C tissue culture incubator with 5.0% carbon dioxide. Ovarian culture medium was αMEM (Gibco) supplemented with 0.23 mM pyruvic acid, 50 mg/L streptomycin sulfate (Biofil), 75 mg/L penicillin (Biofil, China), 0.03 U/mL follicle stimulating hormone (Ningbo, China), and 3 mg/mL bovine serum albumin (Sigma, USA). The ovaries were treated for 24 h by directly adding P7‐, P11‐, and H11‐sEVs or the mTOR inhibitor rapamycin (250 nM, Sigma, USA) to the culture medium. The medium was not changed during the 24‐h treatment period. After the treatment, ovarian section immunofluorescence and ovarian protein western blot experiments were conducted, respectively.

### 4.12 ROS and Mitochondrial Membrane Potential Measurement and Mitochondrial Morphology and Distribution Detection

To determine the reactive oxygen species (ROS) content, mitochondrial membrane potential, and mitochondrial morphology and distribution in hUCMSCs, the procedures were carried out according to the instructions of the ROS detection kit (50101ES01, Shanghai Yisheng Biotechnology Co. Ltd.), JC‐1 detection kit (40706ES60, Shanghai Yisheng Biotechnology Co. Ltd.), and mitochondrial tracer (40740ES50, Shanghai Yisheng Biotechnology Co. Ltd.). Firstly, the cell supernatant was aspirated, and the cells were immediately washed three times with basal medium. Then, complete medium containing staining solution was added, and the cells were incubated in the dark at 37°C for 30 min. After that, the cells were washed with basal medium to remove the excess staining solution. All the viable cells were observed by laser scanning confocal microscope (LSM800, Zeiss, Germany). The relative fluorescence intensity was calculated by ZEN software.

### 4.13 Immunohistochemistry

Tissues from mice were fixed in 10% buffered formalin for paraffin embedding and sectioning. After deparaffinization and rehydration, the sections underwent blocking of endogenous peroxidase activity and antigen retrieval pretreatment. Immunohistochemical analyses were performed using a Histostain Kit (856,743, Invitrogen) with antibodies overnight at 4°C. Diaminobenzidine (DAB) reagent was utilized for coloration on the second day. Non‐immune immunoglobulin G (IgG) was applied as a negative control. The antibodies used are listed in Table S1.

### 4.14 Immunofluorescence and TUNEL Assay

Tissues were fixed with 4% paraformaldehyde (PFA) at 4°C for 24 h. Tissues were dehydrated in a gradient of 10%, 20%, and 30% sucrose, embedded in OCT (4583, SAKURA), and then frozen sectioned. After cryosectioning at 5 μm, the samples were washed three times with pre‐cooled PBS. Then, the tissues or cells were stained with primary antibodies according to the instructions. After completion of primary antibody incubation, the incubation was continued with a fluorescently labeled secondary antibody. Nuclei were stained with Hoechst33342. The confocal microscopic pictures were processed using the confocal microscope (LSM 800, Zeiss, Germany). The antibodies used are listed in Table S1. Terminal uridine nucleotide end labeling. TUNEL analysis was done using a standard kit according to the manufacturer's instructions (40307ES60, Yeasen, Shanghai, China) and viewed under a laser scanning confocal microscope, and relative fluorescence intensity was quantified via ZEN software.

### 4.15 Superovulation

Three mice per group were injected intraperitoneally with PMSG, euthanized 48 h later, and oocytes were collected from both ovaries.

### 4.16 Mitochondrial Membrane Potential Measurement

To detect the mitochondrial membrane potential of oocytes, the experiment was performed following the instructions of TMRE mitochondrial membrane potential assay kit (Cat. No. C2001S, Beyotime Biotechnology, China). Briefly, GV oocytes were isolated from the ovaries and washed three times with M2 medium. Subsequently, oocytes were incubated in M2 medium supplemented with TMRE working staining solution at 37°C under dark conditions for 30 min. After incubation, excess staining reagent was removed by washing oocytes with M2 medium. Fluorescence signals of live oocytes were captured with a laser scanning confocal microscope (LSM800, Zeiss, Germany), and relative fluorescence intensity was quantified via ZEN software.

### 4.17 Immunoblotting

Analysis Proteins were extracted from sEVs or cells by using radioimmunoprecipitation assay (RIPA) lysis buffer (P0013B, Beyotime Institute of Biotechnology) contained protease inhibitor cocktails (M221, Amresco, USA). Protein quantification was performed using the BCA protein quantification kit (BI‐WB005‐500 T, Biochannel, Nanjing, China). A total of 10 μg of protein from each sample was loaded and separated via electrophoresis (165‐8000, Bio‐Rad, USA). The proteins were then electronically transferred to polyvinylidene fluoride membranes. The membranes were blocked in 5% skimmed milk‐TBST (TBS containing 0.1% Tween 20) for 60 min and incubated overnight at 4°C with specific antibodies. After washing the membranes with Tris‐buffered saline with Tween 20 (TBST) three times, horseradish peroxidase (HRP)‐conjugated secondary antibodies were used to detect the proteins via enhanced chemiluminescence (RPN2232, GE Healthcare, USA) on the Tanon 5200 analysis system. The antibodies and conjugated secondary antibodies are listed in Table S1.

### 4.18 Quantitative Real‐Time PCR

Total RNAs from sEVs and cells were purified using the TRIzol reagent (Invitrogen, USA) according to the manufacturer's protocol. The RNA concentrations were measured using a spectrophotometer (NanoDrop 2000c, Thermo Scientific, USA). Subsequently, the FastQuant RT kit (Tiangen Biotech, China) was used to generate cDNA from the RNA. The target gene transcripts were detected using SYBR Green mix (Applied Biological Materials, Canada) on an ABI StepOnePlus platform (Thermo Scientific, USA), following the manufacturer's protocols. Quantification of various mRNAs was performed using the actin amplification signal as a control. The relative expression of the target genes was measured using the 2‐ΔΔCT method. All of the primer sequences used are listed in Table S2. MiRNA levels were measured using the BulgeLoop miRNA qPCR Primer Set (RiboBio, Guangzhou, China) as per the manufacturer's instructions.

### 4.19 Proteomics Analysis

sEV samples of P7‐sEV, P11‐sEV, and H11‐sEV were collected and submitted to Shanghai Bioprofile Biotechnology Co. Ltd. (China) for quantitative proteomic analysis. Differentially expressed proteins (DEPs) were screened based on the combination of *p*‐value and fold‐change (FC) thresholds. Proteins with *p*‐value < 0.05 and FC ≥ 1.5 or FC ≤ 1/1.5 were defined as significantly differentially expressed proteins.

### 4.20 Statistical Analysis

GraphPad Prism 8.0 was used to perform the Student's *t*‐test or two‐way ANOVA to compare the statistical differences between experimental groups. ImageJ (NIH, USA) was used to analyze western blot protein bands. All data are presented as mean ± SEM. *p* < 0.05 was considered to be statistically significant.

## Author Contributions

Yating Chen and Wei Liu contributed equally to this work. Yating Chen and Jing Li conceived and designed the study, supervised all experiments, and drafted the initial manuscript. Yating Chen, Wei Liu, and Ying Tian performed animal experiments, collected and analyzed data, and wrote the methodology section. Yating Chen, Wei Liu, Ying Tian, Xinyu Wang, Haonan Fan, and Mingyu Yang contributed to data analysis, figure preparation, and manuscript formatting. Jing Li and Feiyang Diao participated in the experimental design and provided administrative support. Jing Li and Feiyang Diao supervised the project, provided administrative and financial support, and approved the final manuscript. All authors have read and agreed to the final content of this manuscript.

## Funding

This work was supported by the National Natural Science Foundation of China (U24A20659).

## Conflicts of Interest

The authors declare no conflicts of interest.

## Supporting information


**Figure S1:** Characterization of hUCMSCs. (A) Expression profiles of surface markers CD90, CD73, CD105, CD19, CD11b, CD45, CD34, CD31, and HLA—DR in hUCMSCs at passage 11. (B)Alizarin red staining of calcium nodules and Oil Red O staining of lipid droplets in cells of passages P7, P8, P9, P10, and P11. Scale bar = 50 μm (left), Scale bar = 100 μm (right).(C) RT‐qPCR quantification of relative CDKN1A and CDKN2A senescence‐associated mRNA expression in P7–P11 and H11 cells. (D) Western blot detection of SIRT1 and DNA damage marker γH2A.X protein levels in P7, P11, and H11 cells.(E) Quantitative densitometric analysis of relative SIRT1 and γH2A.X protein expression. Data are expressed as mean ± SEM. *p < 0.05, **p < 0.01, ***p < 0.001, ns, no significance.

**Figure S2:** Detection of Mitochondrial Function in hTERT—UCMSCs. (A) Tom20 mitochondrial immunofluorescence staining of P7–P11 and H11 hUCMSCs; red: Tom20, blue: Hoechst nuclear staining, white dashed boxes indicate magnified mitochondrial regions. (B) RT‐qPCR detection of relative mRNA expression of M1 markers (IL‐6, CD80) and M2 markers (IL‐10, Arg1) in macrophages with different interventions. (C) Western blot detection of characteristic sEV proteins CD9, CD63, Calnexin and HGS in 217‐sEVs. 217‐sEVs refer to P11‐sEVs that have been treated with antagomir‐217‐5p. (D) Nanoparticle tracking analysis (NTA) of sEVs, showing particle size distribution and concentration. Data are expressed as mean ± SEM. *p < 0.05, ***p < 0.001, ns, no significance. Scale bar = 20 μm.

**Table S1:** Antibodies list.

**Table S2:** Primer sequence.

## Data Availability

The data that support the findings of this study are available from the corresponding author upon reasonable request.
