# Supplementary material for: hTERT Immortalization Stabilizes Human Umbilical Cord MSCs and Maintains Their Small Extracellular Vesicles With Preserved Immunomodulatory Activity and Primordial Follicle‐Activating Capacity
Source: Aging Cell. 2026 Aug 21;25(9):e70681. doi: 10.1111/acel.70681 (PMC13495773; doi:10.1111/acel.70681)
Supplement: Supplementary file 1 [file ACEL-25-e70681-s001.docx]

**Figure S1: Characterization of hUCMSCs.**

Figure S1: Characterization of hUCMSCs. (A) Expression profiles of surface markers CD90, CD73, CD105, CD19, CD11b, CD45, CD34, CD31 and HLA - DR in hUCMSCs at passage 11. (B)Alizarin red staining of calcium nodules and Oil Red O staining of lipid droplets in cells of passages P7, P8, P9, P10, and P11. Scale bar = 50 μm(left), Scale bar = 100 μm(right).(C) RT-qPCR quantification of relative CDKN1A and CDKN2A senescence-associated mRNA expression in P7–P11 and H11 cells .(D) Western blot detection of SIRT1 and DNA damage marker γH2A.X protein levels in P7, P11 and H11 cells.(E) Quantitative densitometric analysis of relative SIRT1 and γH2A.X protein expression .Data are expressed as mean ± SEM. *p < 0.05, **p < 0.01, ***p < 0.001, ns, no significance.
