# Supplementary material for: hTERT Immortalization Stabilizes Human Umbilical Cord MSCs and Maintains Their Small Extracellular Vesicles With Preserved Immunomodulatory Activity and Primordial Follicle‐Activating Capacity
Source: Aging Cell. 2026 Aug 21;25(9):e70681. doi: 10.1111/acel.70681 (PMC13495773; doi:10.1111/acel.70681)
Supplement: Supplementary file 2 [file ACEL-25-e70681-s002.docx]

**Figure S2: Detection of Mitochondrial Function in hTERT – UCMSCs.**

Figure S2: Detection of Mitochondrial Function in hTERT – UCMSCs. (A) Tom20 mitochondrial immunofluorescence staining of P7–P11 and H11 hUCMSCs; red: Tom20, blue: Hoechst nuclear staining, white dashed boxes indicate magnified mitochondrial regions. (B) RT-qPCR detection of relative mRNA expression of M1 markers (IL-6, CD80) and M2 markers (IL-10, Arg1) in macrophages with different interventions. (C) Western blot detection of characteristic sEV proteins CD9, CD63, Calnexin and HGS in 217-sEVs. 217-sEVs refer to P11-sEVs that have been treated with antagomir-217-5p. (D) Nanoparticle tracking analysis (NTA) of sEVs, showing particle size distribution and concentration. Data are expressed as mean ± SEM. *p < 0.05, ***p < 0.001, ns, no significance. Scale bar = 20 μm.
