# Supplementary material for: hTERT Immortalization Stabilizes Human Umbilical Cord MSCs and Maintains Their Small Extracellular Vesicles With Preserved Immunomodulatory Activity and Primordial Follicle‐Activating Capacity
Source: Aging Cell. 2026 Aug 21;25(9):e70681. doi: 10.1111/acel.70681 (PMC13495773; doi:10.1111/acel.70681)
Supplement: Supplementary file 3 [file ACEL-25-e70681-s003.doc]

Table S2

Primer sequence

| Gene Name | sense sequence | antisense sequence |
| --- | --- | --- |
| *Gapdh* | GCGGGCGCTGGAGGAA | GGATCTTCATGAGGTAGTCA |
| *Il-6* | ACTCAC CTC TTC AGA ACG AAT TG | CCATCTTTGGAAGGTTCAGGT TG |
| *Arg-1* | CCAGAAGAATGGAAGAGTCAGTGT | GCAGATATGCAGGGAGTCACC |
| *Il-1α* | CGAAGACTACAGTTCTGCCATT | GACGTTTCAGAGGTTCTCAGAG |
| *IL-10* | GACTTTAAGGGTTACCTGGGTTG | TCACATGCGCCTTGATGTCTG |
| *CD80* | ATACGACTCGCAACCACAC | ATGATGACAACGATGACGAC |

| Gene Name | sense sequence | antisense sequence |
| --- | --- | --- |
| *Il-1β* | AGATGCCTGAGATACCCAAAACC | CCAAGCACACCCAGTAGTCT |
| *MMP-3* | TTAAAGACAGGCACTTTTGGCG | CCCTCGTATAGCCCAGAACT |
| *GAPDH* | GAAGGTGGTGAAGCAGGCGT | ATGTGGGCCATGAGGTCCACCA |
| *hTERT* | GCGTTTGGTGGATGATTTCT | CAGGGCCTCGTCTTCTACAG |
| *CDKN1A* | TGTCCGTCAGAACCCATGC | AAAGTCGAAGTTCCATCGCTC |
| *CDKN2A* | CCCAACGCACCGAATAGTTA | ACCAGCGTGTCCAGGAAG |
