# Supplementary material for: hTERT Immortalization Stabilizes Human Umbilical Cord MSCs and Maintains Their Small Extracellular Vesicles With Preserved Immunomodulatory Activity and Primordial Follicle‐Activating Capacity
Source: Aging Cell. 2026 Aug 21;25(9):e70681. doi: 10.1111/acel.70681 (PMC13495773; doi:10.1111/acel.70681)
Supplement: Supplementary file 4 [file ACEL-25-e70681-s004.doc]

Table S1

Antibodies List

| Antibody Name | Manufacturer | Item number |
| --- | --- | --- |
| PE-conjugatedCD11b | BD | 561689 |
| FITC-conjugatedCD19 | BD | 560994 |
| FITC-conjugatedCD34 | BD | 560942 |
| FITC-conjugatedCD44 | BD | 560977 |
| FITC-conjugatedCD45 | BD | 560976 |
| PE-conjugatedCD73 | BD | 561014 |
| PE-conjugatedCD90 | BD | 561970 |
| APC-conjugatedCD105 | BD | 562408 |
| FITC-conjugatedHLA-DR/DQ | BD | 555563 |
| β-ACTIN | CST | 3700 |
| GAPDH | CST | 5174 |
| CD63 | Proteintech | 67605-1-Ig |
| HGS | Santacruz biotechnology | sc-271455 |
| Calnexin | Abcam | ab22595 |
| Alix | Proteintech | 124422-1-AP |
| CD9 | Proteintech | 20597-1-AP |
| rpS6 | Cell Signaling Technology | 2217 |
| P-rpS6(S235/236) | Cell Signaling Technology | 2211 |
| mTOR | Cell Signaling Technology | 2983 |
| P-mTOR | Cell Signaling Technology | 5536 |
| AKT | Cell Signaling Technology | 2920 |
| P-AKT | Cell Signaling Technology | 4060 |
| Foxo3a | Cell Signaling Technology | 12829 |
| iNOS | Proteintech | 60291 |
| p21 | Proteintech | 10355-1-AP |
| P16 | Zembio | R23424 |
| TERT | Santa | Sc-393013 |
| PCNA | Cell Signaling Technology | 13110S |
| SIRT1 | OriGene | TA809834S |
| COX IV | Cell Signaling Technology | 11967S |
| γH2A.X | Abcam | ab81299 |
| Tom20 | Cell Signaling Technology | 42406S |
| PE-Cy7-conjugatedF4-80 | Biolegend | 123113 |
| FITC-conjugatedCD11c | Biolegend | 117305 |
| APC-conjugatedCD206 | Biolegend | 141707 |
| Anti-rabbit IgG | Cell Signaling Technology | 7074 |
| Anti-mouse IgG | Cell Signaling Technology | 7076 |
